# Omega 3-DHA and Delta-Tocotrienol Modulate Lipid Droplet Biogenesis and Lipophagy in Breast Cancer Cells: The Impact in Cancer Aggressiveness

**DOI:** 10.3390/nu11061199

**Published:** 2019-05-28

**Authors:** Nathalia Pizato, Larissa Fernanda Melo Vasconcelos Kiffer, Beatriz Christina Luzete, José Antonio Fagundes Assumpção, Luis Henrique Correa, Heloisa Antoniella Braz de Melo, Lívia Pimentel de Sant’Ana, Marina Kiyomi Ito, Kelly Grace Magalhães

**Affiliations:** 1 Department of Nutrition, University of Brasilia, UnB, Brasilia 70910-900, Brazil; pizatonat@unb.br (N.P.); larissa.vasconcelos94@gmail.com (L.F.M.V.K.); biachristinaa@gmail.com (B.C.L.); 2 Laboratory of Immunology and Inflammation, Department of Cell Biology, University of Brasilia, UnB, Brasilia 70910-900, Brazil; zeassumpcao@gmail.com (J.A.F.A.); henrique.costacorrea@gmail.com (L.H.C.); heloisa.antoniella@gmail.com (H.A.B.d.M.); liviapsdourado@gmail.com (L.P.d.S.); marinakito@gmail.com (M.K.I.)

**Keywords:** docosahexaenoic acid (DHA), delta-tocotrienol (Delta-T3), lipid droplets, lipophagy, breast cancer

## Abstract

Omega 3-docosahexaenoic acid (DHA) and vitamin E Delta-tocotrienol (Delta-T3) are extensively studied as protective nutrients against cancer development. Little is known about the biological mechanisms targeted by these bioactive molecules on lipid droplet (LD) biogenesis, an important breast cancer aggressiveness marker, and the occurrence of lipophagy in breast cancer cells. The aim of this study was to investigate the effect of DHA, Delta-T3 and DHA plus Delta-T3 co-treatment in LD biogenesis and lipophagy process in triple negative breast cancer cell line MDA-MB-231. Cells were treated with 50 μM DHA and/or 5 μM Delta-T3. Our results demonstrated that DHA can trigger an increase in LD biogenesis and co-treatment with Delta-T3 was able to reduce this LD biogenesis. In addition, we showed that a higher cytoplasmic LD content is associated with a higher breast cancer cells malignance and proliferation. Reduction of cytoplasmic LD content by silencing ADRP (adipose differentiation-related protein), a structural LD protein, also decreased cell proliferation in MDA-MB-231 cells. Treatment with DHA and Delta-T3 alone or co-treatment did not reduce cell viability. Moreover, we showed here that DHA can trigger lipophagy in MDA-MB-231 cells and DHA plus Delta-T3 co-treatment was able to enhance this lipophagy process. Our findings demonstrated that co-treatment with DHA plus Delta-T3 in MDA-MB-231 cells could reduce LD biogenesis and potentiate lipophagy in these cells, possibly having a positive impact to inhibit breast cancer malignancy. Therefore, suitable doses of DHA and Delta-T3 vitamin E isoform supplementation can be a prominent tool in therapeutic treatments against breast cancer.

## 1. Introduction

Tumor cells often require increased production of metabolic intermediates for proteins and lipids synthesis as compared to healthy tissues, being a prerequisite for a rapid proliferation [1]. Currently, there is a consensus about critical roles that cellular lipid metabolism exerts on energetic homeostasis of tumor cells, including an association of higher expression of genes that belong to lipid anabolism and catabolism pathways with malignance phenotype [2,3]. In this context, lipid droplets (LD) have been widely studied as an important organelle in cancer biology. LD are intracellular organelles that store neutral lipids and participate in inflammatory mediator’s synthesis [4]. These organelles play a key role in harmonizing lipid trafficking for different cellular activities, especially providing a substrate for beta-oxidation and membrane synthesis [5]. An increase in cytoplasmic LD number in cancer cells is associated with higher aggressiveness and poor prognostic in several types of cancer such as breast cancer [6,7], colon cancer [8,9] and prostate cancer [10]. In a process known as lipophagy, where the degradation of stored lipids occurs, fatty acids can be mobilized from LD [11] and fuel cancer cells. Recently a positive association between cell aggressiveness and increased LD content has been reported in different cancer cell lines, among them breast cancer [12].

Breast cancer is, currently, the most common cancer among women in the world. This type of cancer is a heterogeneous disease highly modulated by hormones signaling, depending on the expression of the estrogen receptor (ER), progesterone receptor (PR), and human epithelial receptor 2 (HER2). Breast cancer can be classified into luminal A, luminal B, HER2 positive, and triple-negative subtypes (TNBC) [13]. It has been showed that TNBC patients have worse overall survival than non-TNBC patients [10]. Among several cell lines widely used for breast cancer studies, we can highlight MCF-7 which are ER positive, PR negative and HER2 positive cells and MDA-MB-231 which are triple negative cells [14].

There is a higher percentage of mortality related to TNBC compared to other types of breast cancer [15,16]. Lipid metabolism in these TNBC cells is more activated, so lipophagy is used as an energy homeostasis maintenance mechanism, and its modulation is poorly described in the literature in cancer context [17]. Since LD play a central role on the lipophagy process and it is increased in the most aggressive type of cancers, it is important to better understand how LD can be modulated under different conditions of cancer adjuvant treatments.

LD biogenesis and lipophagy can be important events for adjuvants treatments against breast cancer. In the past few years, a considerable number of researches exploring tumor modulation proprieties of omega-3 molecules and vitamin E as coadjutants treatments have been increasing, but specific mechanisms involved in this process are still poorly understood.

The family of polyunsaturated omega 3 fatty acids has been widely described as beneficial agents in several cellular processes, including a prominent anti-tumor activity, particularly involving Docosahexaenoic Acid (DHA 22: 6n-3) [18,19,20,21]. DHA was successfully used as an adjuvant capable of increasing the efficacy of other anticancer agents, with no observed adverse effects [22]. Despite that, it is important to emphasize that DHA plays a role in lipid metabolism and possible will modulate differentially diverse cell types according to the impact of this energy pathway for intracellular activities [23]. Moreover, vitamin E supplementation has been used to reduce breast tumor development [24,25,26] and vitamin E Tocotrienols isoforms showed superior antioxidant, anticancer, anti-inflammatory, cardioprotective and neuroprotective properties when compared to tocopherols isoforms [27,28,29,30]. However, the effect of DHA and vitamin E co-treatment is still weakly investigated in cancer research. It has been described that DHA, in the presence of Delta-Tocotrienol (Delta-T3), is able to enhance apoptotic cell death in TNBC cell lines and downregulate carcinogenic parameters [31].

Since LD biogenesis and lipophagy can be considered tumor aggressiveness biomarkers and indicators of higher carcinogenic activity, we aimed to evaluate the effects of DHA and Delta-T3 co-treatment in LD biogenesis and lipophagy in MDA-MB-231 cells. This study is the first to note that DHA supplementation can increase LD biogenesis in breast cancer cells and this event may be related to increased breast cancer aggressiveness. Moreover, DHA plus Delta-T3 co-treatment could reduce LD biogenesis in breast cancer cells, possibly due to Delta-T3 antioxidant properties. We also verified that DHA-treated cells showed a significant increase in lipophagy. In addition, co-treatment with Delta-T3 enhanced the lipophagy event in these cells.

## 2. Materials and Methods

### 2.1. Cells and Treatment

MDA-MB-231 cells were cultured in Minimum Essential Medium (MEM) supplemented with 10% fetal bovine serum (FBS), 1% sodium pyruvate, 1% non-essential amino acid mixture, 1% L-glutamine, 100 units/mL penicillin and 100 μg/mL streptomycin. MCF-7 cells were grown in Dulbecco’s modified Eagle’s medium (DMEM; Gibco; Thermo Fisher Scientific, Inc., Waltham, MA, USA) supplemented with 10% FBS, 100 U/mL penicillin and 100 µg/mL streptomycin (all from Gibco; Thermo Fisher Scientific, Inc.). MCF10A cells were cultured in DMEM /F12 Ham’s mixture supplemented with 5% Equine Serum (Gemini Bio, West Sacramento, CA, USA), EGF 20 ng/mL (Sigma-Aldrich, St Louis, MO, USA), insulin 10 μg/mL (Sigma-Aldrich, St Louis, MO, USA), hydrocortisone 0.5 mg/mL (Sigma-Aldrich, St Louis, MO, USA), cholera toxin 100 ng/mL (Sigma-Aldrich, St Louis, MO, USA), 100 units/mL penicillin and 100 μg/mL streptomycin. All cell cultures used in the present study were obtained from Rio de Janeiro Cell Bank (RJCB), a certified repository of cell lines, and tested for Mycoplasma detection. The VenorGeM^®^ Mycoplasma Detection Kit (Sigma-Aldrich, St Louis, MO, USA, Catalog Number MP0025) was employed for the Mycoplasma PCR-based assay and all cell cultures used in the present work were authenticated by short tandem repeat [STR] profiling [32,33].

In all experiments the cells MCF-7, MCF-10A and MDA-MB-231 were treated with docosahexaenoic fatty acid (DHA—50 μM) and Delta-Tocotrienol (Delta-T3—5 μM), isolated or associated in co-treatments. These concentrations were used because they are considered physiological for the cells and showed no damaging effect on cell viability [34,35].

### 2.2. Assay of Aggressiveness—Cell Transfection with Short Interfering (si)RNA—ADRP

ADRP silencing knockdown in MDA-MB-231 cells was obtained following the protocol described by Shen and colleagues [36]. Briefly, MDA-MB-231 cells were plated (5 × 10^5^) into 6-well plates and grown to 50% confluence. After 24 h, cells were transfected with 25 nM (final concentration) of siGENOME non-targeting siRNA2, human ADRP siGENOME SMART pool (Thermo Fisher Scientific, Inc., Waltham, MA, USA) using Dharma FECT1 transfection reagent, according to the manufacturer’s protocol (Thermo Fisher Scientific, Inc., Waltham, MA, USA). Following 24 h of incubation, the transfection medium was replaced with complete medium. The efficiency of siRNA for ADRP silencing experiment was assessed by staining cells with Bodipy or a guinea pig anti-human ADRP polyclonal antibody (Research Diagnostics Inc. Flanders, NJ, USA) and analyzing by flow cytometry, as well as by western blotting.

### 2.3. Western Blot

MDA-MB-231 cells were transfected with 25 nM (final concentration) of siGENOME non-targeting siRNA2, human ADRP siGENOME SMART pool (Thermo Fisher Scientific, Inc., Waltham, MA, USA) using Dharma FECT1 transfection reagent, according to the manufacturer’s protocol (Thermo Fisher Scientific, Inc., Waltham, MA, USA). Following 24 h of incubation, the transfection medium was replaced with complete medium. MDA-MB-231 cells were collected on ice, washed twice with PBS, lysed with lysis buffer, and centrifuged at 12,000× g for 10 min at 4 °C. The cell lysate was heated at 100 °C for 5 min, and the protein content was determined by BCA assay (Sigma-Aldrich, St Louis, MO, USA). The same amount of proteins was loaded to a 10% SDS-PAGE. Proteins were then transferred to PVDF membrane (Pall Corporation, Ann Arbor, USA) and blocked with 5% skim milk for 2 h. The membranes were probed with primary antibodies against b-Actin (Abcam, Cambridge, USA) and a guinea pig anti-human ADRP polyclonal antibody (Research Diagnostics) at 4 °C overnight. Later, the primary antibodies were washed away with TBST for 1 h and the membranes were treated with HRP-coupled secondary antibodies (Promega Corp., Madison, USA) for 1 h, and washed with TBST afterwards. Finally, Detection of each protein was performed using the ECL kit (Abcam plc, 330 Cambridge Science Park, Cambridge UK).

### 2.4. Assay of Cytotoxicity

The MDA-MB-231 cells were plated in a density of 1 × 10^5^ cells/well and grown for 24 h. The various concentrations of DHA (0, 12, 5, 25, 50, 100 and 200 μM) and Delta-T3 (2.5, 5, 10, 20 and 40 μM) or their co-treatments were added for 24 h, the untreated control received only 0.2% dimethyl sulfoxide (DMSO) (solvent). Cell viability was performed using 3-(4,5-Dimethyl-thiazol-2-yl) -2,5-diphenyl-tetrazolium bromide (MTT) assay in triplicate. MDA-MB-231 cells were seeded in a 96-well plate at a density of 1 × 10^4^ cells/well and allowed to adhere overnight. After that, the culture medium was removed and it was added 200 μL of fresh culture medium per well. Thereafter the cells were incubated for 3 h in L15 culture medium with 10% of MTT. The culture medium was removed and 100μL DMSO was added to the wells for 5 min, the solution was transferred for the new plate of 96 wells for reading. Absorbance was measured using spectrophotometer (SpectraMax) at 570 nm absorbance.

### 2.5. Production of Reactive Oxygen Species (ROS)

Intracellular reactive oxygen species (ROS) were measured using CellROX Deep Red and (Sigma-Aldrich, St Louis, MO, USA) according to the manufacturer’s instructions. After 24 h of treatments with DHA, Delta-T3 or their co-treatments, MDA-MB-231 cells were incubated with 5 µM CellROX for 30 min, protected from light at 37 °C. The cells were washed 3 times with phosphate-buffered saline (PBS) and incubated at 4 °C, Fluorescence intensity was measured in the flow cytometry (FACS Verse) in the FL2 channel. The ROS generation was expressed as mean fluorescence intensity.

### 2.6. Lipid Droplet Biogenesis Analysis by Flow Cytometry

The LD biogenesis was quantitated by flow cytometry. The MDA-MB-231 cells were plated in 24 well plates and incubated overnight to adhere to the plate. The cells were treated with DHA, Delta-T3 or their co-treatments for 24 h. The cells were dissociated with trypsin (GIBCO), washed with PBS and incubated with the 4,4-Difluoro-1,3,5,7,8-Pentametil-4-Bora-3′,4′-Diaza-S-Bodipy (Bodipy 492/595) (Sigma-Aldrich, St Louis, MO, USA) in a stock solution of 1 mg/mL in PBS. It was used 1:7000 Bodipy work solution in PBS (*v*/*v*). The cells were incubated with this solution for 30 min at 4 °C in the dark. The cells were washed 2 times with PBS, resuspended in 500 μL of PBS and stored at 4 °C until reading by FACS Calibur using the FL1 channel.

### 2.7. Lipid Droplet Biogenesis Analysis by Confocal Microscopy

The MDA-MB-231 cells were plated in 24-well plates with a round coverslip in each well, the plates were stored overnight at 37 °C in order to cells adhere in the round coverslip. After the cells were treated with DHA, Delta-T3 or their co-treatments for 24 h, cells were washed once with PBS and fixed with paraformaldehyde (4%) for 10 min. Next, they were washed three more times with PBS. Next, the cells were incubated with Bodipy dissolved in PBS at 1:300 (*v*/*v*), for 30 min at room temperature in the dark. The cells were washed 3 times with PBS. Then cells were washed three times with PBS, and 300 μL of 4 ‘solution, 6-diamidino-2-phenylindole (DAPI) at 1:5000 in PBS (*v*/*v*) was added and the cells were incubated with this solution for 5 min. Then they were washed 3 times with PBS. The round coverslips with the cells were fixed on the microscope slide with prolong according to the manufacturer’s (Invitrogen, Thermo Fisher Scientific, Inc., Waltham, MA, USA) instructions. The images of the LD were obtained by confocal microscopy (Leica TCS SP5 fluorescent microscopy). The capture of images by Leica TCS SP5 fluorescent microscopy was made with an increase of 63× and zoom of 4.

### 2.8. Lipophagy Analysis

The lipophagy in MDA-MB-231cells was characterized by the identification of the co-localization of LC3-B (which stains autophagosome) and Bodipy (which stains LD). Antibodies anti-LC3-B (Invitrogen, Thermo Fisher Scientific, Inc., Waltham, MA, USA) was used as primary antibody and Alexa Fluor 456 (Invitrogen, Thermo Fisher Scientific, Inc., Waltham, MA, USA) as a secondary antibody. The cells were plated in 24-well plates with a round coverslip in each well. The plates were stored at 37 °C overnight to allow cells to adhere in the round coverslip. Then the cells were treated with DHA, Delta-T3 or their co-treatments for 24 h, and they were washed with PBS once and fixed with paraformaldehyde (4%) for 10 min. The cells were washed three times with PBS and fixed for permeabilization with triton (0.2%) diluted in PBS for 20 min. Next, they were washed three times with PBS and were incubated with blocking buffer for 20 min. The blocking buffer was removed and the primary antibody LC3-B was added to the wells at 1:500 *v*/*v* in blocking buffer and remained in contact with the cells at 4 °C in the dark overnight. The cells were washed three times with PBS and incubated with secondary antibody Alexa fluor 456 at the dilution of 1:2000 (*v*/*v*) and the Bodipy probe dilution at 1:300 (*v*/*v*) in PBS for 60 min at room temperature in the dark. The cells were washed 3 times with PBS and incubated for 5 min at room temperature with DAPI diluted in PBS at 1:5000 (*v*/*v*). Next, the cells were washed three times with PBS. Samples stained only with the secondary antibody were used as an experimental control. The round coverslips with the labeled cells were fixed on the microscope slide with prolong (Invitrogen, Thermo Fisher Scientific, Inc., Waltham, MA, USA) according to manufacturer’s instructions. The lipophagy was observed by confocal microscopy (Leica TCS SP5 fluorescent microscopy). The capture of images by Leica TCS SP5 fluorescent microscopy was made with an increase of 63× and zoom of 4.

### 2.9. Clonogenic Assay

The ability of MDA-MB-231 cells to form colonies was assessed by the clonogenic assay as described by Rafehi and colleagues [10]. The MDA-MB-231 cells suspension were prepared by trypsinization. Cells were washed with phosphate buffered saline and incubated with a 0.05% trypsin/EDTA solution for 5–10 min. Trypsin was neutralized with Dulbecco’s modified eagle medium containing 10% fetal bovine serum. The cells were detached by pipetting up and down (20 times). Cells were plated in 6-well plate in a humidified stove at 37 °C, the number of cells in each well were carefully counted using a Neubauer chamber with trypan blue staining, and were diluted in an appropriate number of cells (500 cells per well), to achieve ~90% confluency on the day of the experiment. Cells were treated with DHA, Delta-T3 or co-treatments for 24 h. After the treatment, the culture medium was removed and replaced by complete L15 culture medium with L-glutamine, FBS (10%), and antibiotic/antimycotic (1%). This culture medium was changed every 3 days until 14 days of cultivation, which it is accepted that the time must be equivalent to at least six cell divisions. After this period the cells were fixed as methanol and acetone at 1:1 *v*/*v* during 20 min, and stained with 5 mL 0.01% (*w*/*v*) crystal violet in dH_2_O for 60 min. The excess crystal violet was washed with dH_2_O and allow dishes to dry. Digital images of the colonies were obtained using a camera device, and colonies were counted using imaging analysis software packages ImageJ (Fiji Version 1.44a). GraphPad Prism software was used for statistical analysis.

### 2.10. Cell Migration Assay—Wound Healing Assay

The cells were plated in 24 well plates. After reaching confluence it was made a risk using one pointer of 100 µl and a ruler. The wells were washed with complete L15 culture medium. The cells were treated with DHA, Delta-T3 or their co-treatments for 24 h. During treatments, the culture plates were photographed at different times (0 h, 16 h and 24 h) in bright microscope [37,38]. The analysis of the results was performed with the ImageJ software and the risk closure area was measured using the formula: (Initial area − end area) / (Initial area) × 100 = the closing percentage of area [39].

### 2.11. Statistical Analysis

For the analysis, we used one-way ANOVA for multiple comparisons and the Tukey post-test. The results were expressed as differences in the mean of the values compared to untreated control cells. For the analysis, the statistical program Graph Pad Prism 5.00 (Trial version) was used.

## 3. Results

### 3.1. MDA-MB-231 Cell Line Aggressiveness

**Figure 1:**
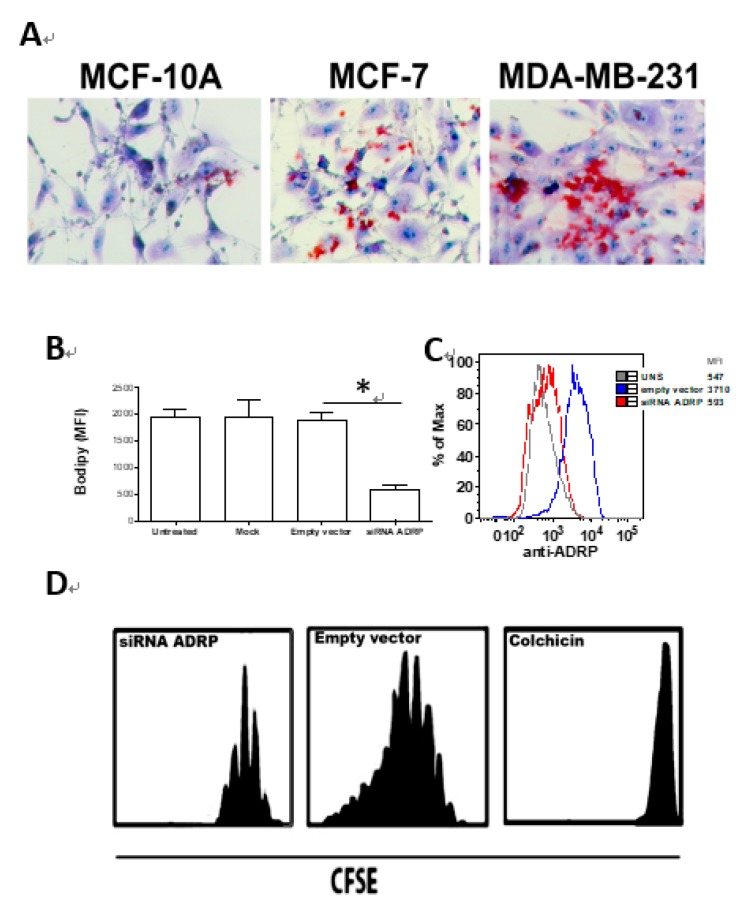
(**A**). Lipid droplet biogenesis in MCF-10A, MFC-7 and MDA-MB-231 was assessed by Oil Red O staining and analyzed by bright microscopy, magnification of 40×. (**B**). Lipid droplet biogenesis of MDA-MB-231 cells treated with siRNA for adipose differentiation-related protein (ADRP) silencing was assessed by Bodipy staining and analyzed by flow cytometry. (**C**). ADRP expression of MDA-MB-231 cells treated with siRNA for ADRP silencing was assessed by immunostaining of cells with anti-ADRP and analyzed by flow cytometry, numbers represent mean fluorescence intensity (MFI), statistical significance is represented by an asterisk with *p* < 0.05. (**D**). Cell proliferation of MDA-MB-231 cells treated with siRNA for ADRP silencing was assessed by Carboxyfluorescein Succinimidyl Ester (CFSE) staining and analyzed by flow cytometry. Histograms are representative of three independent experiments.

LD biogenesis was analyzed in MCF-10A, MFC-7 and MDA-MB-231 cells as showed in Figure 1A. Our results showed differential LD biogenesis among all these three cell lineages. Non-malignant MCF-10A cells presented less LD cytoplasmic content compared to the highly malignant MDA-MB-231 cells, while MCF-7 cells were found to present an intermediate amount of cytoplasmic content. Considering the elevated amount of LD in MDA-MB-231 cells, a siRNA for ADRP silencing was used in order to knock-down ADRP expression and lead to impaired LD formation and ADRP expression by flow cytometry (Figure 1B,C) or western blotting (Figure S1). After that, cell proliferation was analyzed using CFSE staining (Figure 1D). Results showed a higher cell proliferation capacity in MDA-MB-231 cells treated with empty vector compared to those ones treated with siRNA for ADRP silencing, suggesting LD biogenesis play an important role in this breast cancer cell proliferative capacity.

### 3.2. Determination of DHA, Delta-T3 and Co-Treatment Cytotoxicity

**Figure 2:**
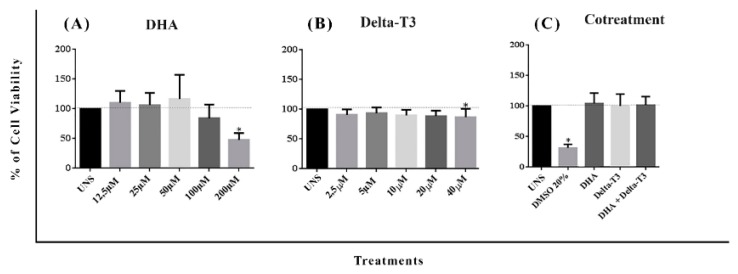
(**A**). Cytotoxicity of DHA at concentrations of 12.5 μM, 25 μM, 50 μM, 100 μM and 200 μM. (**B**). Cytotoxicity of delta-tocotrienol (Delta-T3) at concentrations of 2.5 μM, 5 μM, 10 μM, 20 μM and 40 μM. (**C**). Cytotoxicity of DHA (50 μM) plus Delta T3 (5 μM) co-treatment. All MDA-MB-231 cells were treated for 24 h and cytotoxicity was measured by MTT (*n* = 5). Values were expressed as mean ± SD. Results considered statistical had *p* < 0.05 (*) compared to unstimulated MDA-MB-231 cells (UNS).

For subsequent analysis, it was established, based on a cytotoxicity assay with a range of concentrations, that 50 µM and 5 µM were considered as non-toxic physiological concentrations for DHA and Delta T3 vitamin E, respectively. Only cells treated with DHA at 200 µM presented a significant decrease in cell viability as shown in MTT assay in Figure 2A. Neither Delta-T3 nor co-treatment with DHA plus Delta-T3 showed any impact in MDA-MB-231 cells viability in doses analyzed here (Figure 2B,C).

### 3.3. Reactive Oxygen Species (ROS) Production

**Figure 3:**
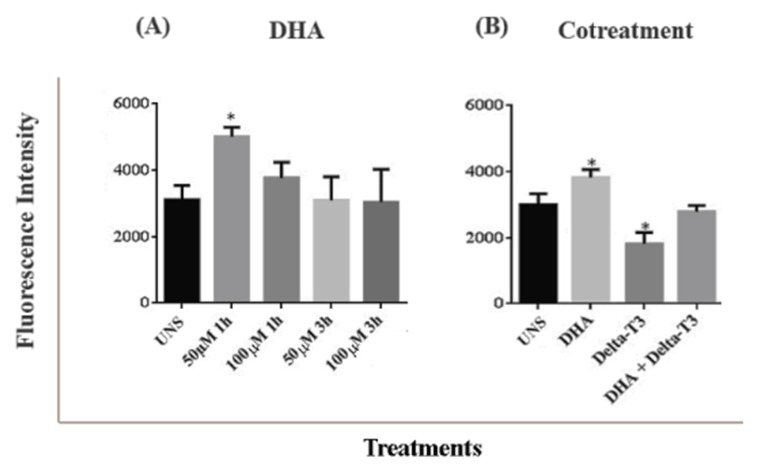
(**A**) Reactive oxygen species (ROS) generation in MDA-MB-231 cells treated with DHA for 1 or 3 h (50 μM and 100 μM). (**B**) ROS generation in MDA-MB-231 cells treated with DHA (50 μM), Delta-T3 (5 μM) and co-treatment for 1 h. ROS generation was assessed by cell ROX deep red staining (*n* = 3). Values expressed in mean ± SD. Results considered statistical had *p* < 0.05 (*) compared to unstimulated cells (UNS).

Treatment with DHA at 50 μM for 1 h showed a significant increase in ROS generation compared to the unstimulated cells as showed in Figure 3A. However, other concentrations of DHA in different period of incubation time did not trigger ROS increased generation.

Delta-T3 treatment showed an opposite effect to DHA treatment, reducing ROS generation when compared to unstimulated cells (UNS) (*p* < 0.05) as showed in Figure 3B. Co-treatment with DHA plus Delta-T3 for 1 h showed no difference when compared to unstimulated cells or cells treated only with DHA or Delta-T3.

### 3.4. Lipid Droplet Biogenesis

**Figure 4:**
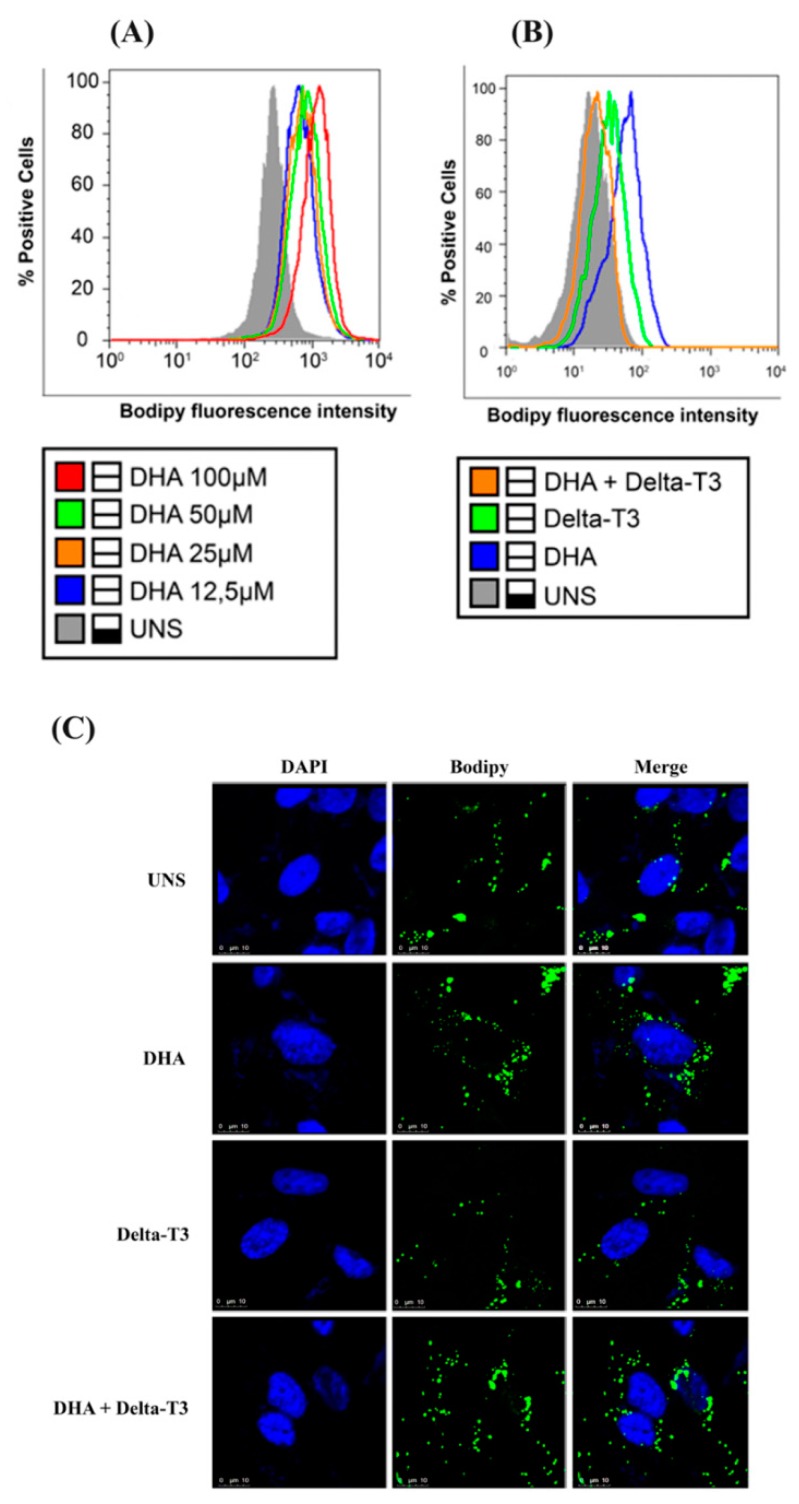
(**A**) Lipid droplet biogenesis induced by treatment with Docosahexaenoic acid (DHA) (12.5 μM, 25 μM, 50 μM and 100 μM) for 24 h in MDA-MB-231 breast cancer cells was assessed by Bodipy staining and analyzed by flow cytometry. (**B**). Lipid droplet biogenesis induced by treatment with DHA (50 μM), Delta-T3 (5 μM) and co-treatment was assessed by Bodipy staining and analyzed by flow cytometry. (**C**) Confocal microscopy images of lipid droplet biogenesis (Bodipy staining: green) after treatment of MDA-MB-231 cells with DHA (50 μM), Delta-T3 (5 μM) or co-treatment. Cell nuclei are shown in blue (DAPI staining). Histograms and images are representative of three independent experiments.

LD biogenesis in MDA-MB-231 breast cancer cells was increased in response to DHA treatment, in a dose-dependent manner as showed in Figure 4A. In all concentrations tested, the mean fluorescence intensity of Bodipy staining was increased when compared to unstimulated cells (UNS). Treatment with DHA induced higher LD content compared to unstimulated cells. Treatment with Delta-T3 alone showed only a slight increase in LD content when compared to unstimulated cells. Co-treatment with DHA and Delta-T3 reduced LD biogenesis when compared to cells treated with DHA or Delta-T3 alone as showed both in MDA-MB-231 cells (Figure 4B) and 4T1 cells (Figure S2). Qualitative analysis of these results with MDA-MB-231 cells is also shown by confocal microscopy (Figure 4C).

### 3.5. Lipophagy Assay

**Figure 5:**
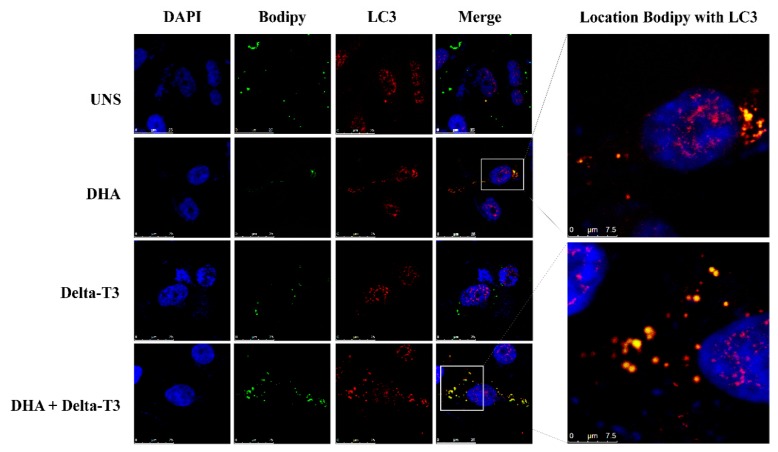
Lipophagy process analyzed in MDA-MB-231 cells treated with DHA (50 μM), Delta-T3 (5 μM) and co-treatment assessed by confocal microscopy images (63×). LD are shown in green (Bodipy staining) and LC3-B is shown in red. Cell nuclei are shown in blue (DAPI staining). Images are representative of three independent experiments.

Our results showed the occurrence of lipophagy in MDA-MB-231 breast cancer cells triggered by DHA treatment by pointing out the co-localization of bodipy and LC3-B staining in these cells. White box indicates zoomed images showing the co-localization between Bodipy staining (green) and LC3-B (red), suggesting active lipophagy in these cells. Treatment with Delta-T3 (5 μM) alone did not induce lipophagy. However, Delta-T3 was able to increase lipophagy event in MDA-MB-231 breast cancer cells as shown in co-treatment condition (DHA plus Delta-T3) in Figure 5.

### 3.6. Clonogenic and Cell Migration

**Figure 6:**
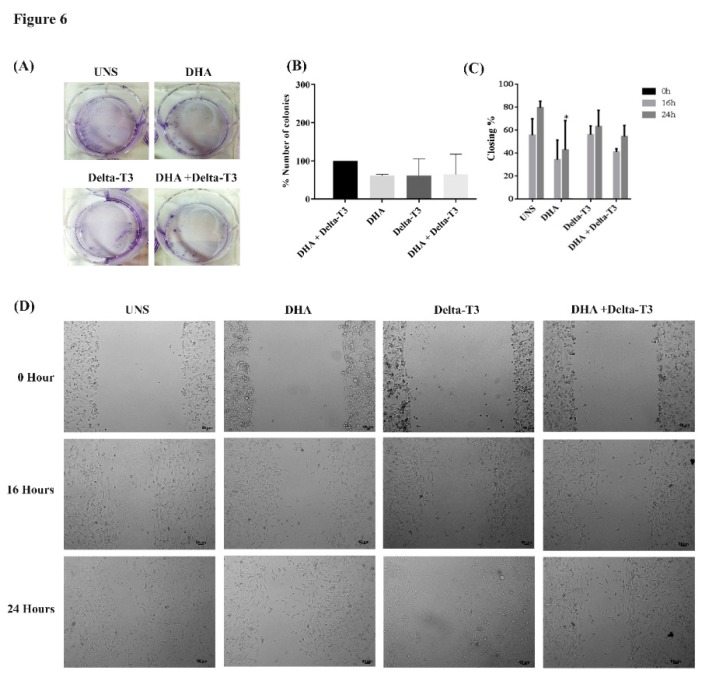
Clonogenic and cell migration. (**A**) Images and (**B**) percentage of the number of MDA-MB-231 colonies treated with DHA (50 μM), Delta-T3 (5 μM) and co-treatment were assessed by clonogenic assay. Cell migration was analyzed by wound healing assay and quantified in percentage (**C**) and representative images at 0, 16 and 24 h (*n* = 3) (**D**). Images are representative of three independent experiments.

Colony forming cell assay (clonogenic) was used to analyze the impact of treatment of MDA-MB-231 cells with DHA, Delta-3T and both together in the cell ability to form cell colonies, an important carcinogenic parameter. Treatment with DHA, Delta-T3 or co-treatment did not alter the MDA-MB-231 cells ability to form cell colonies as shown in Figure 6A,B. In addition, the cell migration ability of MDA-MB-231 cells was assessed by wound healing assay. Treatment with DHA alone at 50 μM for 24 h inhibited cell migration ability when compared to unstimulated MDA-MB-231 cells. Treatment with Delta-T3 (5 μM) did not alter cell migration or modulate DHA effect when used in co-treatment as shown in Figure 6C. Representative wound healing assay images after 0, 16 and 24 h of different treatments were presented in Figure 6D.

## 4. Discussion

This study observed that DHA at 50 μM and Delta-T3 at 5 μM did not affect cell viability but were able to increase LD biogenesis in MDA-MB- 231 TNBC cells. We have previously demonstrated that DHA at 50uM does not modulate this breast cancer cell viability [35]. However, co-treatment of MDA-MB-231 cells with DHA (50 μM) and Delta-T3 (5 μM) together triggered a reduction in LD biogenesis when compared to treatment with DHA alone. This suggests that DHA and Delta-T3, when used together, can modulate LD biogenesis in MDA-MB-231 breast cancer cells, which could have an impact on breast cancer aggressiveness.

TNBC has a complex biology and do not respond to hormonal therapy medicines [40], which can help decrease or even stop the growth of breast cancer cells. This type of cancer presents a poorer prognosis scenario when compared to other breast cancer types. Thus, studies focusing on the investigation of its molecular mechanisms and description of bioactive molecules that could help in adjuvant clinical treatments, influencing tumor growth, could have major importance in breast cancer therapy. It is well known that DHA is beneficial for inhibiting breast tumor carcinogenesis by triggering breast cancer cell death at 100 to 200 μM doses in vitro [35,41]. However, our present data showed that DHA at 50 μM can also induce an increased amount of LD biogenesis in MDA-MB-231 breast cancer cells and that higher LD cytoplasmic content can be correlated with higher cancer aggressiveness.

The aggressiveness of MDA-MB-231 TNBC cells was evaluated considering cell proliferation ability and LD biogenesis since this cell line presents a higher LD content when compared to non-tumorigenic and other tumorigenic breast cells. The role of LD in MDA-MB-231 breast cancer cells metabolism was demonstrated by silencing messenger ribonucleic acids (mRNAs) of Adipose Differentiation-Related Protein (ADRP), an important structural LD protein crucial for LD accumulation and formation. Our results show that knocking-down ADRP mRNA decreases LD biogenesis MDA-MB-231 in breast cancer cells, leading to a considerable reduction in the proliferative capacity of these cells. This observation suggests that an important carcinogenic parameter, such as cell proliferation, in this cancer cells can be altered through LD modulation, highlighting the role of these organelles in breast cancer cells aggressiveness.

Our data showed that the higher the cytoplasmic LD content the higher the cancer aggressiveness. LD amount was shown to correlate with the degree of aggressiveness from the non-malignant MCF10A cells to the highly malignant MDA-MB-231 cells, while MCF-7 cells were found to be intermediately aggressive, which is in accordance with other studies [6,7,42]. However, Wright and colleagues observed an opposite phenomenon where CUB-domain-containing protein 1 (CDCP1) knockdown could increase LD abundance and reduce TNBC 2D migration in vitro and metastasis in vivo [43]. These might be explained by the fact that proteins involved in the CDCP1 pathway, such as Src and PKCδ kinases, are also involved in lipid metabolism as well promoting fatty acid oxidation and subsequent oxidative phosphorylation providing energy and contributing to migration and metastasis. In the present work, our results indicated that malignant breast cancer cells can diminish the TNBC MDA-MB-231 ability to proliferate by decreasing the expression of the LD structural protein ADRP. Cancer aggressiveness is also related to higher LD content in other tumor cells [9,44,45,46,47,48,49] and has been associated with cell invasiveness, and higher resistance to chemotherapy [50,51]. One plausible explanation is that LD can be used as energy substrates for further cellular proliferation, consequently increasing disease progression. Cancer cells are able to enhance lipogenesis and cholesterol production as well as to uptake a larger amount of lipids and to increase fatty acids β-oxidation. In addition, both de novo lipogenesis and upregulation of lipolysis from intracellular storages translate in increased fatty acids availability which favors the transformation of cells and increasing pathogenesis of cancer [7]. LD quantification in tumor cells has become an emerging tool for monitoring tumor aggressiveness in response to treatment in different cancer cell types and could contribute to a better understanding of LD biogenesis mechanisms.

It has been shown that in order to prevent nutrient stress and promote proliferation, breast cancer cells import free fatty acids (FFAs) to either generate energy through β-oxidation or subsequently store them into LD when fatty acids are present in excess inside cells [52]. Excessive FFAs can be cytotoxic to the cells, so LD may provide a cytoprotective mechanism to store these intracellular fatty acids [53,54,55], decreasing cell death caused by lipotoxicity [42,56,57]. DHA supplementation was able to increase the LD biogenesis, similarly to other studies showing increased LD biogenesis in colorectal cancer cell lines after treatment with DHA [58]. This effect, however, was observed in three times higher concentrations than the ones used in our study, suggesting that DHA has a higher potential for cytotoxicity in breast cancer cells, even in reduced concentrations.

DHA is known for inducing ROS production in MDA-MB-231 breast cancer cells [31]. LD biogenesis can be stimulated by an increase in oxidative stress resulting from a higher ROS production [54]. After one-hour treatment with DHA (50 μM), MDA-MB-231 cells showed increased ROS production, an effect that was not observed with three hours of treatment. This is probably due to MDA-MB-231 redox capacity [57]. Treatment with Delta-T3 showed reduced ROS production when compared to unstimulated cells, probably due to its powerful antioxidant action [27,29]. In addition, tocotrienols have the highest antioxidant activity amongst vitamin E isoforms [59,60,61,62]. In our findings, Delta-T3 treatment reduced DHA-induced ROS production.

Usually, tumors are known to have altered metabolism and a higher proliferation rate when compared to normal cells. Therefore, an increased energy supply is demanded in tumor cells and a proposed mechanism to provide energy substrate is autophagy [63,64]. Autophagy presents different roles depending on the cell’s metabolism. Under normal conditions, it maintains cellular homeostasis. In cancer cells, it shows a tumor suppressor activity through the elimination of the oncogenic proteins substrates, toxic proteins and damaged organelles [65]. This pathway is also related to LD which can release stored fatty acids to be used in the cellular metabolism [66]. Taking this into consideration, cancer cells could generate energy substrates through recycling intracellular LD, a process known as lipophagy [67,68].

There is little data available about the correlation among cancer, lipophagy and the tumor microenvironment. Dupon and colleagues studied the role of LD in cervical cancer cells (HeLa) treated with oleic monounsaturated fatty acid and observed an increase in LD biogenesis and autophagy, indicating lipophagy occurrence in the absence of nutrient deprivation [69]. This finding suggests that lipophagy may occur as a homeostatic pathway to control the storage of lipids and lipolysis. Furthermore, lipophagy can supply the cell’s phospholipids demand, facilitating the formation of autophagosome, an essential step for triggering lipophagy [70,71,72]. Most of the studies regarding lipophagy include nutrient-deprived microenvironments, suggesting that higher LD biogenesis as a mechanism through which cells can regulate their energy substrate, maintaining their homeostasis [54,73,74]. In our study model, there was no nutrient deprivation. For this reason, this does not interfere or modulate lipophagy. Treatment of MDA-MB-231 cells with DHA increased lipophagy when compared to unstimulated cells in a nutrient-rich environment, suggesting that DHA probably was used as a lipid substrate for LD biogenesis. Other fatty acids can behave similarly to DHA, such as oleic acid (18:9 n-1), a monounsaturated fatty acid that triggered lipophagy in mammary epithelial cells, hepatocytes, and osteosarcoma cells [75,76].

As well as DHA, studies regarding Tocotrienol (Vitamin E) isoform have revealed a new horizon for this molecule as an antitumor agent [77,78,79], presented as anti-proliferative [80,81], and pro-apoptotic effects in different cancer cell lines [25]. The Tocotrienol isoforms act as antioxidants and anti-inflammatories agents [25,26,27,28,29,30,82]. Here, we have shown that co-treatment of the MDA-MB-231 breast cancer cells with DHA plus Delta-T3 notably reduced LD biogenesis when compared to cells treated with DHA alone, suggesting that Tocotrienol isoforms could modulate DHA-related effects in LD biogenesis. There are few studies evaluating the effects of Tocotrienol isoforms in intracellular lipid metabolism [83,84]. Therefore, there is little information about the mechanisms of action of these isoforms in LD biogenesis. In hepatocellular cancer cells, treatment with Delta-T3 (10–15μM) inhibited intracellular triglycerides accumulation, leading to decreased LD biogenesis [80]. In the preadipocyte cell line (non-tumoral), treatment with Delta-T3 (25μM) also decreased LD biogenesis [59]. Our study used lower concentrations of these bioactive molecules (5 μM), which were still able to reduce LD biogenesis when used together with DHA.

Cellular lipid storages, such as LD, are also targeted for lysosomal degradation via lipophagy, which also occurs in cancer cells [67,85]. Here we demonstrated that DHA treatment can trigger lipophagy in MDA-MB-231 breast cancer cells and co-treatment with Delta-T3 could potentiate this lipophagy event, by showing increased colocalization of LD and LC3-B. This increased lipophagy could explain the reduction of LD biogenesis induced by co-treatment of DHA plus Delta-T3. Other studies also showed increased autophagy as a regulator of lipid metabolism in MDA-MB-231 cells supplemented with Tocopherol (22%) and Tocotrienol (78%) when compared to unstimulated cells [86].

Cell survival of breast cancer cells under treatments was also analyzed, showing no difference between all treatments compared to treated cells. This is probably due to the fact that the concentrations used in this study were based on what we considered to be physiological concentrations. Loss of MDA-MB-231 cells viability after treatment with DHA was only induced cell cytotoxicity at 100 μM. A similar event was observed with Delta-T3, which only induced cell cytotoxicity at 40 μM. Cell migration of MDA-MB-231 cells was reduced by DHA treatment when compared to unstimulated cells, and treatment with Delta-T3 did not show significant differences when compared to treatment with DHA alone. Other studies have reported that treatment with DHA was able to reduce carcinogenic parameters in human hepatocellular carcinoma cells, breast cancer cells, prostate cancer cells, leukemic cells, colonocytes, human colon cancer cells and pancreas cells [87,88,89,90,91,92,93,94,95], which corroborates our findings. Xiong and colleagues showed that co-treatment with DHA and Gamma-Tocotrienol (γT3) induces apoptotic events in TNBC cells [31], but no studies until now analyzed the effect of treatment with Delta-T3 isoform on cell migration.

The effect of the co-treatment with bioactive molecules DHA and Delta-T3 in LD biogenesis and lipophagy in MDA-MB-231 breast cancer cells is poorly understood. Our results showed that despite the differences in LD biogenesis and occurrence of lipophagy, no significant change was seen in the carcinogenesis parameters analyzed in MDA-MB-231 cells with co-treatments. The increased LD content of MDA-MB-231 breast cancer cells reported in this study suggests that the interaction between DHA and Delta-T3 needs to be further investigated. Since increased LD content and lipid accumulation observed in this breast cancer cells treated with DHA at the doses analyzed here are associated with aggressive behavior in MDA-MB-231 cells [96], the doses of the DHA and vitamin E during supplementation of breast cancer patients should be analyzed carefully. We have previously demonstrated that higher doses of DHA have an anti-tumor effect against breast cancer cells MDA-MB-231 cells by triggering proptosis cell death [35].

We believe that higher concentrations of DHA and Delta-T3, as well as a long time treatment, should be tested analyzing the lipophagy event in breast cancer cells. Different concentrations could provide more meaningful changes in carcinogenic parameters since Tocotrienol is classically presented as an antitumor agent. Inflammatory pathways should also be further investigated in this context, considering the importance of inflammation in the tumor microenvironment, cancer lipid metabolism and tumor progression. Besides, it is important to note that in the present manuscript we have only mainly focused on the TNBC MDA-MB-231 cells but it is necessary to consider to investigate this lipid metabolism modulation induced by DHA and Delta-T3 in other breast cancer cell lines such as MB468 and HCC70, and organoids as well in future works.

## 5. Conclusions

DHA and Delta-T3 are largely studied as potentials bioactive molecules that could have an influence on tumor growth reduction, mainly being used as treatment associated with conventional therapies. However, the role of DHA and Delta-T3 is still not completely elucidated in this context. These supplements are capable of interfering with cellular metabolism, directly modulating tumor microenvironment.

In this study, our findings demonstrated that co-treatment with DHA plus Delta-T3 in MDA-MB-231 cells could reduce LD biogenesis and potentiate lipophagy in these cells, possibly having a positive impact to inhibit breast cancer malignance. Therefore, suitable doses of DHA and Delta-T3 vitamin E isoform supplementation can be a prominent tool in therapeutic treatments against breast cancer.

## Supplementary Materials

The following are available online at https://www.mdpi.com/2072-6643/11/6/1199/s1, Figure S1: ADRP expression of MDA-MB-231 cells treated with empty vector or siRNA for ADRP silencing was assessed by immunostaining of cells with anti-ADRP and analyzed by western blotting, Figure S2: Lipid droplet biogenesis of TNBC cells (4T1) treated with DHA (50 μM), Delta-T3 (5 μM) and co-treatment was assessed by Bodipy staining and analyzed by flow cytometry.

## Abbreviations

3-(4,5-dimethylthiazol-2-yl)-2,5-diphenyltetrazolium bromide (MTT), 4 ‘solution, 6-diamidino-2-phenylindole (DAPI), 4,4-Difluoro-1,3,5,7,8-Pentametil-4-Bora-3′,4′-Diaza-S-Bodipy (Bodipy), Adipocyte Differentiation-Related Protein (ADRP), Delta-tocotrienol (Delta-T3), Dimethyl Sulfoxide (DMSO), Docosahexaenoic acid (DHA), Dulbecco’s modified Eagle’s medium (DMEM), Fetal Bovine Serum (FBS), Free Fatty Acids (FFAs), Lipid Droplet (LD), Messenger Ribonucleic Acid (mRNA), Micromolar (μM), Microtubule-Associated Proteins 1A/1B Light Chain 3B (LC3-B), Minimum Essential Medium (MEM), Oxygen Reactive Species (ROS), Phosphate-Buffered Saline (PBS), Ribonucleic acid Small interfering (siRNA), Triple Negative Breast Cancer (TNBC), Triple Negative Breast Cancer Cell Line (MDA-MB-231) and Unstimulated Cells (UNS).
