# Supplementary material for: Omega 3-DHA and Delta-Tocotrienol Modulate Lipid Droplet Biogenesis and Lipophagy in Breast Cancer Cells: The Impact in Cancer Aggressiveness
Source: Nutrients. 2019 May 28;11(6):1199. doi: 10.3390/nu11061199 (PMC6627337; doi:10.3390/nu11061199)
Supplement: Supplementary file 1 [file nutrients-11-01199-s001.pdf]

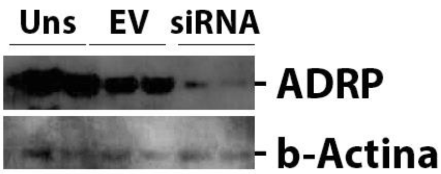


**Figure S1.** ADRP expression of MDA-MB-231 cells treated with empty vector or siRNA for ADRP silencing was assessed by immunostaining of cells with anti-ADRP and analyzed by western blotting.

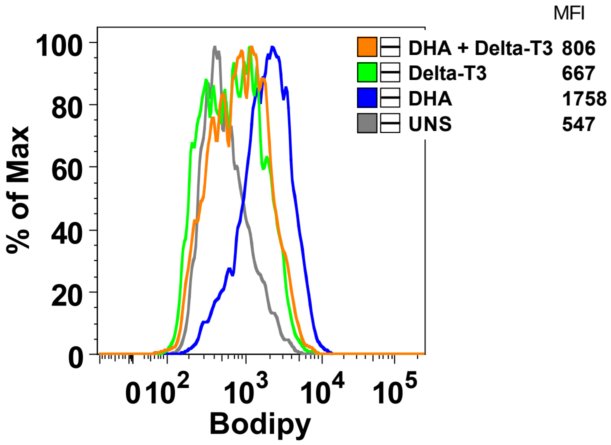


**Figure S2.** Lipid droplet biogenesis of TNBC cells (4T1) treated with DHA (50µM), Delta-T3 (5µM) and co-treatment was assessed by Bodipy staining and analyzed by flow cytometry.
